# The productivity limit of manufacturing blood cell therapy in scalable stirred bioreactors

**DOI:** 10.1002/term.2337

**Published:** 2017-04-03

**Authors:** Rachel Bayley, Forhad Ahmed, Katie Glen, Mark McCall, Adrian Stacey, Robert Thomas

**Affiliations:** ^1^ Centre for Biological Engineering (Holywell Park), Wolfson School of Mechanical and Manufacturing Engineering Loughborough University Loughborough Leicestershire UK

**Keywords:** blood, manufacture, red cell, erythrocyte, culture, bioreactor, cost, productivity

## Abstract

Manufacture of red blood cells (RBCs) from progenitors has been proposed as a method to reduce reliance on donors. Such a process would need to be extremely efficient for economic viability given a relatively low value product and high (2 × 10^12^) cell dose. Therefore, the aim of these studies was to define the productivity of an industry standard stirred‐tank bioreactor and determine engineering limitations of commercial red blood cells production. Cord blood derived CD34+ cells were cultured under erythroid differentiation conditions in a stirred micro‐bioreactor (Ambr™). Enucleated cells of 80% purity could be created under optimal physical conditions: pH 7.5, 50% oxygen, without gas‐sparging (which damaged cells) and with mechanical agitation (which directly increased enucleation). O_2_ consumption was low (~5 × 10^–8^ μg/cell.h) theoretically enabling erythroblast densities in excess of 5 × 10^8^/ml in commercial bioreactors and sub‐10 l/unit production volumes. The bioreactor process achieved a 24% and 42% reduction in media volume and culture time, respectively, relative to unoptimized flask processing. However, media exchange limited productivity to 1 unit of erythroblasts per 500 l of media. Systematic replacement of media constituents, as well as screening for inhibitory levels of ammonia, lactate and key cytokines did not identify a reason for this limitation. We conclude that the properties of erythroblasts are such that the conventional constraints on cell manufacturing efficiency, such as mass transfer and metabolic demand, should not prevent high intensity production; furthermore, this could be achieved in industry standard equipment. However, identification and removal of an inhibitory mediator is required to enable these economies to be realized. Copyright © 2016 The Authors Journal of Tissue Engineering and Regenerative Medicine Published by John Wiley & Sons Ltd.

## 1 Introduction

Blood transfusions are one of the most common clinical interventions worldwide with ~21 million donated blood components transfused each year in the USA alone. Increasing demand due to aging populations, challenges of adventitious agent screening, or requirement for specific immuno‐phenotypes, has created a growing search for alternative sources to public donation. New uses for red blood cells (RBCs) such as targeted drug delivery may increase this demand further (Bourgeaux *et al*., 2016). There is evidence that transfusion of homogenously *young* RBCs may have clinical benefit by decreasing the transfusion frequency of chronically transfused patients (Bosman, 2013; Luten *et al*., 2008). One proposed solution to these issues is the manufacture of RBC from stem or progenitor cells potentially providing an unlimited supply of cells in an optimal age distribution (Zeuner *et al*., 2012).

Anucleate RBCs have successfully been produced *in vitro* from a variety of cell sources including haematopoietic stem cells such as cord blood CD34+ cells, adult mobilised peripheral blood, and bone marrow CD34+ cells (Neildez‐Nguyen *et al*., 2002; Giarratana *et al*., 2005; Miharada *et al*., 2006; Fujimi *et al*., 2008; Giarratana *et al*., 2011). Recently, approaches using human pluripotent cells, both induced and embryonic, have also been reported, although challenges with control of appropriate lineage and development of adult phenotype remain (Qiu *et al*., 2008; Lu *et al*., 2008; Lapillonne *et al*., 2010; Dias *et al*., 2011; Chang *et al*., 2011; Kobari *et al*., 2012). Due to the exceptionally high numbers of erythroblast stage cells required to be maintained in viable culture in any candidate production process, common late stage manufacturing challenges exist irrespective of initial cell source.

Challenges associated with the scale‐up of any cell culture bioprocess include maintaining consistency, quality and quantity of the cell product whilst minimizing cost of production (Rousseau et al., 2014; Timmins and Nielsen, 2009). This is particularly fraught with RBC production due to the requirement for relatively extreme process intensification whilst avoiding detrimental effects on cells, and where there is little understanding of the sensitivities of each stage of the progressively maturing erythroid phenotype to common bioprocess operations. In particular, robust erythroblast enucleation to produce reticulocytes and then fully mature RBCs has been problematic in vitro and the mechanisms still remain to be fully elucidated (Kingsley et al., 2004; Lee et al., 2004). With respect to cost of production, RBC is an example of a high dose product where cost of goods reduction is a priority for commercial viability. It has been estimated that one unit of cultured RBCs would cost $8000–15,000 to produce using current processes, compared to $200–230 for one unit of donated blood (Zeuner *et al*., 2012). The primary reason for this high cost is expensive media components required for *in vitro* differentiation and maturation multiplied by large culture volumes. This has led to calls for research to identify and address the fundamental barriers to efficient production of erythroid cells (Rousseau *et al*., 2014).

Cost effective production of RBCs will require high density cell culture. Conventional culture densities are considered high at 1 × 10^7^ cells/ml, yet this would still require a 200‐l final volume to produce a single unit or 2 × 10^12^ cells. To achieve a final harvest of 2 × 10^12^ cells in a 5‐l volume will require a density of 4 × 10^8^ cells/ml. Neither of these volumes accounts for the production chain to reach the final cell number, or production overage required for cell impurity or cell losses in downstream processing. Clearly there is a need to understand the productivity of RBC manufacture at scale, and the nature of the limitations, to enable the manufactured blood field to move forward. In order to address this, we have used a model system of differentiation of CB CD34+ cells to RBCs in a ml‐scale stirred tank bioreactor system.

It has previously been shown that CB CD34+ cells can proliferate and differentiate to erythroid cells in a scaled down version of industry standard production equipment, the stirred microbioreactor system, Ambr™ (Glen *et al*., 2013; Hsu *et al*., 2012; Ratcliffe *et al*., 2012). In the present study, the intensification limits (bioreactor operation, gas transfer, media usage) of cells in such standard equipment were explored to determine current productivity and limiting mechanisms with respect to key criteria: cost of goods (system volume, media volume per cell and process time per cell) and quality (enucleated cells). This is important to allow the field to take an informed approach to address the engineering and scientific challenges that need to be overcome to generate an economically viable product.

## 2 Materials and methods

Unless otherwise stated, reagents were purchased from Sigma–Aldrich (Dorset, UK).

### 2.1 CD34+ cell culture

Fresh umbilical cord‐derived mononuclear cells were supplied by the Anthony Nolan Cell Therapy Centre (http://www.anthonynolan.org/clinicians‐and‐researchers/cord‐blood‐services) with informed consent and NREC ethical approval. CD34+ cells were isolated via positive selection using CD34 antibody‐labelled microbeads as per the manufacturer's instructions (Miltenyi Biotec, Germany). Mixed donor CD34+ cells (>70% purity) were cryopreserved prior to cell culture. On thaw, CD34+ cells were cultured in accordance with a three‐stage protocol as described previously (Griffiths *et al*., 2012). Briefly, cells were cultured in Iscove's Modified Dulbecco's Medium (IMDM) (Source BioScience) containing 3% (*v*/v) AB Serum (Sigma), 2 mg/ml human serum albumin (Irvine Scientific, USA), 10 μg/ml Insulin (Sigma), 3 U/ml heparin (Sigma), 500 μg/ml iron saturated Transferrin (R&D Systems). In the first stage (days 0–8) this was supplemented with 10 ng/ml SCF, 1 ng/ml interleukin (IL)‐3 and 3 U/ml erythropoietin (EPO); in the second stage (days 8–11) with 10 ng/ml stem cell factor (SCF), 3 U/ml EPO and in the final stage (days 11–20) with 3 U/ml EPO. Cells were cultured in tissue culture flasks at 37°C, 5% CO_2_ for 3 days, after which cells were either maintained in control static culture or transferred to the Ambr bioreactor system (TAP Biosystems, Royston, UK). Bioreactors were preconditioned as described previously (Glen *et al*., 2013) and vessels were gassed either using a sparge tube or via the vessel headspace if nonsparged. 0.1% Pluronic F‐68 (Gibco, Paisley, UK), impeller speed, pH, and O_2_ (percentage of atmospheric), were varied as specified in results.

### 2.2 Culture analysis

#### 2.2.1 Cell count and viability

Online cell counting and viability was measured using a Vi‐Cell XR (Beckman Coulter, USA). Population doublings (PD) were calculated as follows:

$$\begin{array}{l} \mathrm{PDs}=\left\{\left[{\mathrm{LOG}}_{10}\left(\mathrm{CN}/\mathrm{CNi}\right)\right]\times 3.33\right\}\\ \mathrm{CNi}=\text{start cell number},\mathrm{and}\;\mathrm{CN}=\mathrm{end}\;\text{cell number}. \end{array}$$

#### 2.2.2 Flow cytometry of erythroid lineage markers

Cells were sampled to FACS tubes (1 × 10^5^/tube) and incubated with preconjugated antibodies CD34‐FITC (BD Biosciences, San Jose, CA, USA), CD235a‐PE (BD Biosciences) and DRAQ5 (nuclear stain; BioStatus, Loughborough, UK) for 20 min at room temperature (RT). CD235a+/DRAQ5‐ cells were classified as enucleated. Samples were analysed using a BD FACSCanto™ II flow cytometer (BD Biosciences) and gated against specific isotype controls to determine percentage positive cells.

#### 2.2.3 Assessment of cell morphology

Cells (1–4 × 10^5^) were centrifuged at 300 × *g*_av_ for 6 min at RT, supernatant removed, resuspended in 200 μl of medium and centrifuged onto poly‐lysine coated microscope slides (Sigma 3‐16 PK centrifuge with a cytology rotor) at 60 × *g*_av_ for 4 min at RT. Slides were left to air dry overnight, stained using Leishman's stain (VWR International, Radnor, PA, USA) and mounted with mounting medium and a glass coverslip. Slides were examined by bright field microscopy using an Eclipse Ti (Nikon, Tokyo, Japan) at 40× magnification.

#### 2.2.4 High‐performance liquid chromatography for haemoglobin expression

High‐performance liquid chromatography (HPLC) globin chain separation was performed using a protocol modified from Lapillonne *et al*. (2010). Cells (10^6^) were centrifuged at 300 × *g*_av_ for 6 min at RT, lysed in 50 μl water, and stored at –80°C. On thaw, samples were centrifuged at 13,000 × *g*_av_ at 4°C for 10 min and the lysates collected. Supernatant (10 μl) was injected onto a 1.0 × 250 mm C4 column (Phenomenex, Macclesfield, UK) with a 42% to 56% linear gradient between mixtures of 0.1% trifluoracetic acid in water (Buffer A) and 0.1% trifluoracetic acid in acetonitrile (Buffer B) at flow rate of 0.05 mL/min for 50 min (Dionex HPLC Ultimate 3000 system; Thermo Fisher Scientific, Camberley, UK). The column temperature was 50°C and the UV detector set at 220 nm.

#### 2.2.5 Cytokine analysis

Ten analytes [IL‐1β, IL‐2, IL‐4, IL‐6, IL‐10, interferon‐γ, tumour necrosis factor‐α, transforming growth factor (TGF)‐β1, TGF‐β2, and TGF‐β3] were quantified from cell culture supernatant with the Bio‐Plex Pro™ Human Cytokine Group I, 7‐plex assay kit and the Bio‐Plex Pro TGF‐β, 3‐plex assay kit (Bio‐Rad Laboratories, Hercules, CA, USA) according to the manufacturer's instructions. Data were acquired using a Bio‐Plex‐200 suspension array system and concentrations calculated with Bio‐Plex Manager software 6.1 on a Bio‐Plex® MAGPIX™ instrument using a standard curve derived from a recombinant cytokine standard (supplied by the manufacturer).

#### 2.2.6 O_2_ consumption rate

Erythroblasts were taken at a series of time‐points and O_2_ consumption assessed using an O_2_ sensitive phosphorescent probe mixed with cells at 1 × 10^7^/ml in a 96‐well plate format as per manufacturer's instructions (Cayman Chemical, Ann Arbor, MI, USA). A FLUOstar Omega plate reader (BMG Labtech, Orternberg, Germany) recorded ratiometric time‐resolved fluorescence (Excitation =380 ± 20 nm / Emission =650 ± 50 nm) and O_2_ consumption (mg/cell.h) was calculated based on a 0.9% solubility of O_2_ in saline solution at 37°C under 1 atmospheric pressure (6.7 mg O_2_/l). Maximum supportable cell density in commercial scalable systems was calculated using the formula:

$$\text{Cell density}=\mathrm{Kla}\;\left(C^{*}-C\right)/R$$

Where Kla = reported mass transfer coefficient of system (/h), C* = saturation O_2_ concentration (6.7 mg/l), C = maintenance O_2_ concentration (3.35 mg/l), and R = O_2_ consumption (mg/cell.h).

#### 2.2.7 System medium per cell volumetric productivity analysis

Erythroblasts were taken at day 7 of culture and volumetric productivity calculated for cultures seeded in fresh media at 3 × 10^6^/ml, 5 × 10^6^/ml and 5 × 10^6^/ml with 30% of medium replaced after 5 h:

Volumetric medium productivity (volume/cell) = Media volume used/( I.e^rt^ – I).

I (initial cell number), r (growth rate constant, h^*–*1^), (t) time when growth rate becomes inhibited.

Uninhibited growth rate (r) was estimated from an exponential fit to the first 12 points of each high resolution (0.75‐h counts) growth curve; time of growth inhibition (t) was determined as the point at which cell numbers deviated from extrapolation of this uninhibited model.

#### 2.2.8 Media exhaustion studies

Erythroblasts were taken at day 7 of culture, centrifuged at 300 × *g*_av_ for 6 min at RT, and resuspended in fresh culture medium at 3 × 10^6^/ml. Medium and cells were sampled hourly. Controls were cultured without intervention; experimental supplemented concentrations at 10 h were 2250 mg/l glucose, 292 mg/l glutamine, 1.5% AB serum, 5 ng/ml stem cell factor, 0.5 ng/ml interleukin‐3 and 1.5 U/ml erythropoietin alone or in combination as specified in results. Amino acids (MEM Amino acids 50× solution, M5550), vitamins (BME Vitamins 100× solution, B6891) and phosphate (sodium phosphate monobasic, S5011) were supplemented at initial concentrations. Ammonium hydroxide and lactic acid were added to erythroblast cultures at 3 × 10^5^/ml to assess the effect on cell growth (1.3 mm, 8 mm ammonia; 5 mm, 28 mm lactate). Metabolite and nutrients were measured (or verified) using a Cedex Bio HT – Bioprocess Analyser (Roche, Switzerland).

### 2.3 Statistics and calculations

Statistical comparisons and design of experiment statistical design were conducted using Minitab™ software. ANOVA was used to establish P‐values and Tukey's test where pairwise comparisons are stated. A minimum of *n* = 3, was used to power statistical comparisons. Growth rate in the presence of inhibitors was calculated from an exponential fit to a six‐point data series over 18 h. Growth response to supplements was calculated by the rate of deviation from extrapolated uninhibited exponential growth. Where percent enucleation is reported, it is reported to coincide with the peak system proliferation, avoiding misleadingly high percentage enucleation figures that occur as cell numbers decline.

## 3 Results

### 3.1 Erythroblast bioreactor compatibility and cell density intensification limitations

Three cell‐type specific attributes, in combination with the mass transfer characteristics of a bioreactor, determine the cell density that can be supported in a culture system: tolerance to bioreactor operation (and therefore achievable mass transfer), required dissolved O_2_ level, and O_2_ uptake rate (OUR). Given the importance of culture intensification to RBC manufacture, each of these was determined for erythroblast culture.

#### 3.1.1 Tolerance of erythroblast culture to bioreactor agitation and gassing

Mechanical agitation and gas sparging of a cell culture improves mass transfer and therefore O_2_ availability to cells. However, consequent mechanical stress can reduce cell viability or alter phenotype; in the case of erythroid lineage cells impeller tip speeds of >210 mm/s have been reported to be damaging (Chisti, 2001), and gassing can damage cells during bubble rupture. Further, gas damage can be exacerbated by mechanical agitation due to bubble break up and increased bubble to cell surface interface (Chisti, 2000). To test these operational factors, stir speeds of 300 revolutions/min (RPM; 157 mm/s) and 450 RPM (236 mm/s) in combination with O_2_ delivery via sparging through the medium or the reactor headspace were investigated for effects on cell proliferation and erythroblast maturation.

**Figure 1:**
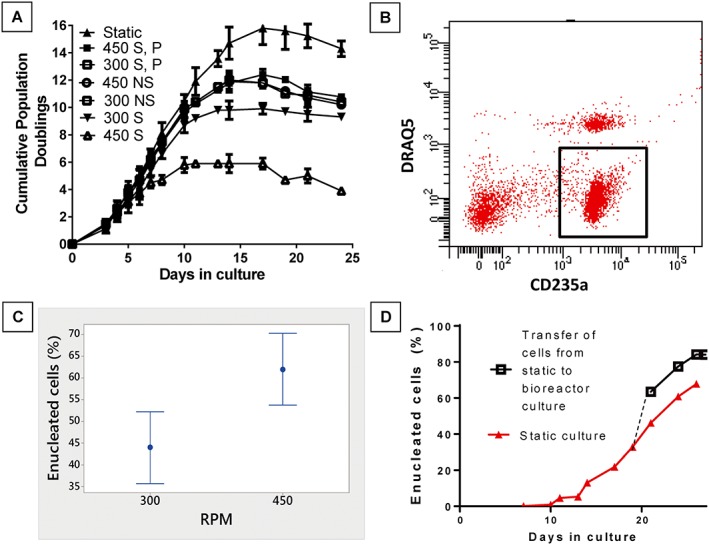
Erythroid cell proliferation and differentiation is affected by bioreactor operational factors that determine system mass transfer. Mechanical agitation, gas sparging and cell protective PF68 were tested for effect on growth and maturation. (A) Growth curves show gas sparging (S) with stirring (300 or 450RPM) greatly reduced cell proliferation; stirring exacerbated the negative effect of sparging but was not detrimental alone (NS). PF68 supplementation to sparged bioreactors (S, P) protected proliferation from mechanical damage and was equivalent to nonsparged bioreactors (NS). (B) Example flow cytometry plot of CD235a vs. the nuclear stain DRAQ5 shows clear identification of the enucleated population (box). (C) In a nonsparged system, higher mechanical agitation supported a higher enucleation rate after 18 days. (D) Mechanical agitation was shown to have a direct effect on enucleation by transfer of cells from static to bioreactor culture after 19 days; the parallel curves indicate this accelerated enucleation is not associated with increased enucleated cell fragility.

Sparged and stirred bioreactors substantially reduced erythroblast proliferation relative to static culture. This effect was increased at higher tip speed with static culture total PDs (TPD) of 15.3, decreasing to 9.9 and 6.0 at 300 and 450 RPM respectively (*p ≤* 0.05). In the absence of sparging, cell proliferation in the bioreactor was improved, but still reduced relative to static culture (*p ≤* 0.05). However, there was no significant difference between the different tip‐speeds (300 RPM, TPD = 12.0, 450 RPM (TPD = 11.9), or any measured reduction in viability, indicating that mechanical damage was unlikely to be the reason for this remaining proliferative deficit in nonsparged bioreactors (Figure 1A). Addition of the nonionic surfactant Pluronic‐F68 (PF‐68) was investigated to mitigate sparging induced damage; PF‐68 restored sparged bioreactor cell growth to the level of nonsparged controls, increasing tolerable O_2_ input rate, and therefore increasing potential cell density (Chisti, 2000; Tharmalingam *et al*., 2008) (Figure 1A). Enucleated RBC production under each condition was evaluated by a flow cytometry assay of CD235a+/DRAQ5– cells (Figure 1B). Although protective of growth, PF‐68 had a negative impact on the percentage of enucleated cells at the end of the process. This negative effect persisted when PF‐68 was removed from the cultures at Day 7 (nonsparged control enucleation =68%, sparged + PF‐68 = 43%, sparged + PF‐68 until Day 7 = 44%; *p ≤* 0.05). In the absence of sparging, a higher tip‐speed generated substantially more enucleated product (Figure 1C). Transfer of cells from static culture to stirred culture after 19 days resulted in a rapid increase in enucleated cells demonstrating this was a direct effect of stirring on enucleation (Figure 1D).

#### 3.1.2 Effect of dissolved O_2_ and pH level on erythroblast culture

The second erythroblast attribute necessary to determine maximum potential cell density is the dissolved O_2_ concentration. Both O_2_ and pH are reported to effect erythroid differentiation (Endo *et al*., 1994; McAdams *et al*., 1998; Sarakul *et al*., 2013); a matrix of pH and O_2_ conditions were investigated in the bioreactor system to determine relative magnitude of effect and independence.

**Figure 2:**
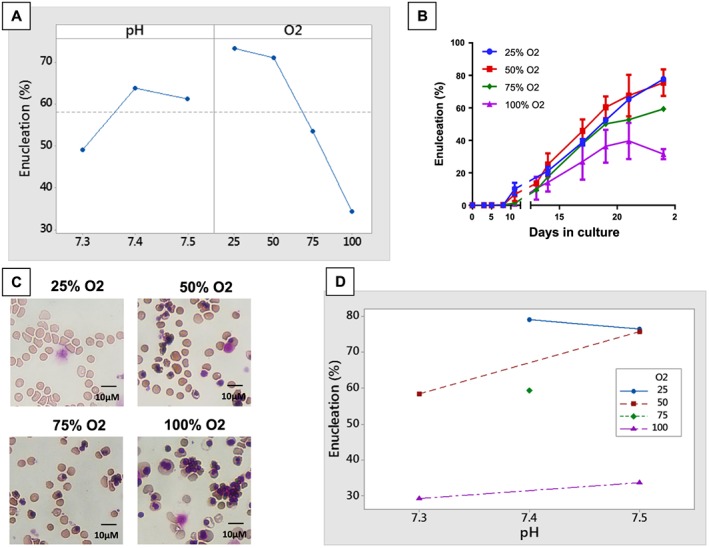
Lower dissolved O_2_ increases the percentage of enucleated cells. (A) Lower dissolved O_2_ increases the percent of the cell population enucleated (*p =* 0.004). 25% and 50% O_2_ form a statistically distinct group from higher O_2_ levels (*p* ≥ 0.05). pH is not a statistically significant factor (pairwise comparison indicates the difference between pH 7.3 and 7.5 close to significance, *p =* 0.14). (B) Cell morphology clearly shows higher enucleation levels at lower O_2_ (Day 19 cells cytocentrifuged, stained with Leishmans dye, observed with a Nikon Eclipse Ti microscope with a 40× objective. (C) The level of enucleation is higher throughout the culture process at low O_2_, not just at final harvest. (D) An interaction chart for pH and O_2_ suggests a rise in percent enucleation with increased pH may be more significant when O_2_ is at an intermediate level.

Lower dissolved O_2_ greatly increased the percentage of enucleated cells (Figure 2A–C). At 25% O_2_ there were 78% enucleated cells, which was significantly higher than the 37% enucleated cells observed at 100% O_2_ (*p ≤* 0.01). pH did not appear to be a significant factor affecting enucleation; however, pairwise comparison showed the difference between pH 7.3 and 7.5 to be close to significance (*p =* 0.14); this is in agreement with the advantage to elevated pH reported previously and our observation of the persistence of non‐CD235a expressing cells at pH 7.3 (data not shown). A rise in the percentage of enucleated cells occurred with increased pH at intermediate level O_2_, indicating sensitivity to pH effect may be greater if dissolved O_2_ is not optimized (Figure 2D). pH and O_2_ had no significant effect on total cell proliferation or time to maximum product yield, with the TPD ranging from 12.0 to 12.6 in all cultures and the maximum product yield achieved between 17 and 20 days.

#### 3.1.3 Comparison of the bioreactor produced cells to a static culture system

**Figure 3:**
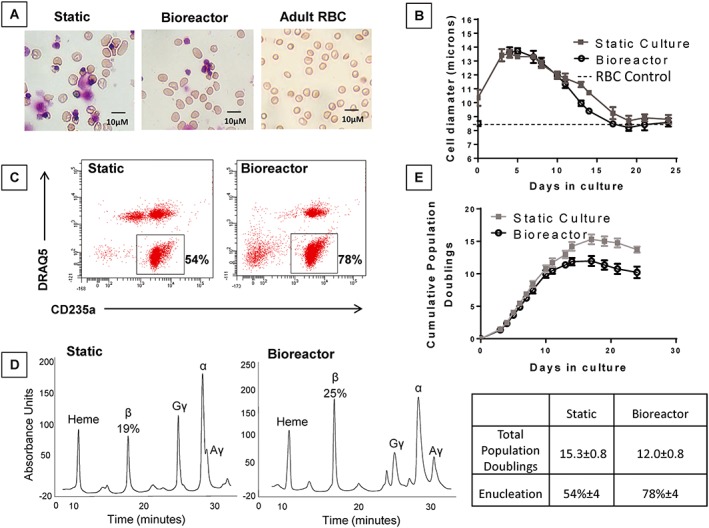
Erythroid cells cultured in the established bioreactor system had reproducibly improved homogeneity but lower total proliferation relative to cells generated in the control static culture system. (A) Photographs of cells from static, bioreactor and a primary control show mature enucleated cells in both systems (cytocentrifuged onto glass slides, stained with Leishmans dye, visualised using a Nikon Eclipse Ti microscope equipped with a 40× objective). (B) The diameter of cells in static and bioreactor cultures show a reduction with time to the size of the primary control red blood cells. This was slightly accelerated in the bioreactor. (C) The percentage of enucleated erythroid cells (CD235a+/DRAQ5–) in bioreactor cultures at the point of peak proliferation was greater than in the control static culture. (D) Haemoglobin status of cells obtained from static and bioreactor cultures after 21 days was similar (HPLC). The percentage indicates the proportion of β haemoglobin chain expression. (E) Cumulative population doublings of cells in static and bioreactor cultures and associated table shows reduced total proliferation in the stirred bioreactor (*n* = 5 independent bioreactor runs from separate primary cell isolations, data is mean ± standard error of the mean).

The established bioreactor process (pH 7.5/50% O_2_/450 RPM/nonsparged) was compared to the control static culture system. After 21 days in culture, a large number of mature enucleated cells were observed in both systems with a similar appearance to the adult donor RBC control (Figure 3A). The mature RBCs cultured *in vitro* were also similar in size to adult RBC (static =8.8 μm, bioreactor =8.3 μm, adult donor control RBC = 8.5 μm; Figure 3B). The percentage of enucleated cells was higher in bioreactor cultures (78 ± 4%) compared to static (54 ± 4%; *p ≤* 0.05; Figure 3C), illustrating that increased homogeneity of enucleated cell product is achieved in the bioreactor system. Analysis of haemoglobin expression showed broad equivalence between static and bioreactor systems, and comparability to other reports from cord cells (Jin *et al*., 2014), including significant expression of β‐globins (Figure 3D). The approximately 3 TPD deficit in proliferation in bioreactor culture relative to static culture was confirmed as previously observed (Figure 3E).

#### 3.1.4 Determining specific O_2_ uptake rate of erythroblasts

**Figure 4:**
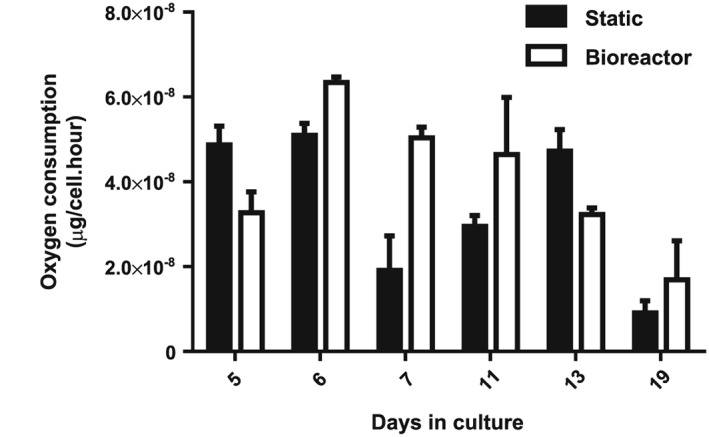
O_2_ is not a factor limiting cell expansion in the Ambr bioreactor system. The specific OUR of erythroblasts at serial time points was monitored via an O_2_ sensitive fluorescent probe. OUR is low relative to conventional cell lines and declines as cells mature

The maximum cell density supportable is determined by the rate of O_2_ transfer into the medium in the established bioreactor process relative to the cells OUR (Xing *et al*., 2009). Cell OUR was monitored throughout the CD34+ to RBC differentiation process. Maximal OUR occurred at Day 6 in both static and bioreactor culture (static =5.10 × 10^–8^ μg O_2_/cell.h and bioreactor =6.34 × 10^–8^ μg O_2_/cell.h; Figure 4). After this point the OUR of cells in the bioreactor declined and reached 1.69 × 10^–8^ μg O_2_/cell.h by Day 19. Cells in static culture had a more variable OUR following Day 6, but this still decreased to 9.11 × 10^*–*9^ μg O_2_/cell.h by Day 19. The known mass transfer characteristics of commercial scale culture systems (Junker, 2004; Klockner *et al*., 2013; Mikola *et al*., 2007; Nienow *et al*., 2013) allows calculation of the density of erythroblasts supportable in the absence of other culture limitations, and the compatibility of those systems with constraints on bioreactor operation to increase mass transfer (identified above; Table 1). Calculations are based on consumption rates of 2.3 × 10^*–*7^ μg O_2_/cell.h to allow a significant (4‐fold) safety margin and indicate that cell densities in excess of 5 × 10^8^/ml (target density to allow a sub‐10 l system volume/unit of 2 × 10^12^ cells) should be supportable in various commercially available bioreactors.

**Table 1:** Calculation of the density of erythroblasts achievable in commercial scaled systems based on mass transfer properties in the absence of other culture limitations (calculated based on 2.3 × 10^*–*7^ μg O_2_/cell.h to allow a 4‐fold safety margin from observed peak OUR); compatibility with erythroblast culture is noted based on the sparging and mechanical sensitivities presented in Figure 1

**System**	**Representative Kla (/h)**	**Theoretical density supported (cells/ml)**	**System compatible with erythroblasts**
Pilot scale to large production sparged stirred tank (1–20 kl)	60–380	1.6 × 10^9^–9.8 × 10^9^	No
10–50 l nonsparged stirred vessels	10–100 (depending on agitation and fill volume)	2.6 × 10^8^–2.6 × 10^9^	Yes
200 l nonsparged (i.e. Cultibag Orb)	10–20	2.6 × 10^8^–5.2 × 10^8^	Potentially
5–10 l wave bag	2–60 (dependent on rocking rate and head space gas exchange)	5.2 × 10^7^–1.6 × 10^9^	Potentially
Ambr bioreactor system	3–5	8.0 × 10^7^–1.3 × 10^8^	Yes

### 3.2 Erythroblast medium volumetric productivity limit

**Figure 5:**
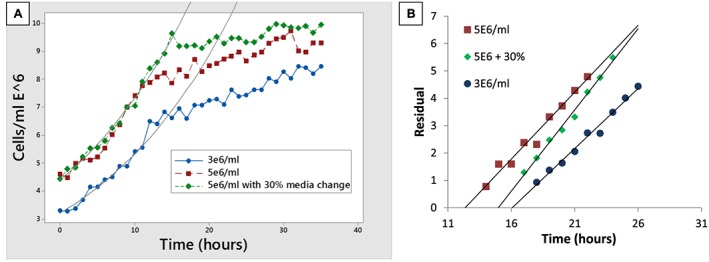
Volumetric productivity of the system is dependent on media exchange strategy (A) Cells were cultured starting at 3 × 10^6^/ml, 5 × 10^6^/ml, and 5 × 10^6^/ml including a 30% volume exchange after 5 h. Cells initially proliferated at a constant and equivalent rate under all conditions after which growth became inhibited. (B) The initial deviation of the cell numbers from the extrapolated exponential growth is approximately linear (R^2^ > 95%), and can be used to approximate the time point at which growth became inhibited.

Given that O_2_ availability was not the primary bioreactor limitation at current culture densities the culture medium utilisation of the system was assessed. Erythroblasts from Day 6 were placed into fresh medium in bioreactors at different densities (3 × 10^6^/ml, 5 × 10^6^/ml) and with an alternate media exchange strategy (5 × 10^6^/ml with 30% exchange after 5 h) to construct high resolution growth curves. Exponential growth models of the first 9 h (12 data points) were all equivalent for growth rate (0.05 1/h) and an excellent fit (R^2^ > 96% in all cases) indicating no significant impact of initial cell density or partial media exchange on growth rate (Figure 5A). Deviation of the data from the extrapolated model identified when growth inhibition occurred; 15.2 h (3 × 10^6^/ml), 12.4 h (5 × 10^6^/ml), 16.1 h (5 × 10^6^/ml with 30% media change after 5 h; Figure 5B). The medium replacement rate per cell produced required to keep erythroblasts in uninhibited growth was strategy dependent suggesting increased productivity from higher density culture (Table 2); this bioreactor protocol would require a lower media volume/unit produced (495 l/unit) compared to the original static laboratory protocol (662 l/unit; Table 2). Further, the bioreactor protocols maintenance of a ~ 13.9 h cell doubling time will only require 58% of the manufacturing facility time relative to the control static process (~24 h doubling) for any given output, with substantial cost implications.

**Table 2:** The number of cells produced up until the time at which growth becomes inhibited can be used to calculate the volumetric productivity of each strategy if uninhibited growth were maintained. *Significantly (*p ≤* 0.05) different from 3 × 10^6^/ml bioreactor

**Condition**	**Start cell density**	**Growth rate (h)**	**Doubling time (h)**	**Vol (l)/unit**
Static Protocol	1.00 × 10^6^	0.029	24.25	662
Bioreactor	3.00 × 10^6^	0.051	13.93	573
Bioreactor	5.00 × 10^6^	0.051	13.93	*495
Bioreactor (30% 5 h media exchange)	5.00 × 10^6^	0.051	13.93	*501

### 3.3 Screening of factors limiting medium volumetric productivity

**Figure 6:**
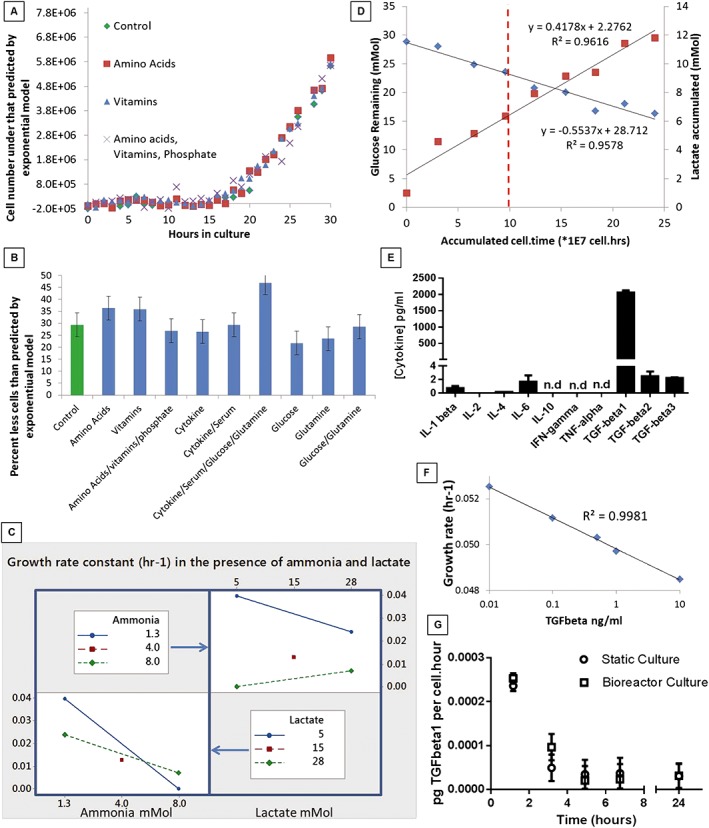
Depletion of medium factors or production of common metabolites and cytokines are not responsible for volumetric productivity limits. (A) As previously, the deviation of cell growth from the initial exponential rate can be plotted as exponential model residuals vs. time. Supplementation after 10 h with amino acids, vitamins, or amino acids, vitamins and phosphate do not change the point at which growth becomes inhibited. (B) A wider range of supplementation strategies were tested including combinations of cytokines, serum, glucose and glutamine. The percent reduction in cells at 24 h compared to that predicted by the exponential model for each strategy is shown indicating no support of additional cell growth relative to control for any supplementation strategy. (C) Ammonia and lactate both inhibit cell growth (*p* ≤ 0.05). An increase in lactate concentration reduces the inhibitory effect of ammonia at high levels of the latter. (D) Lactate accumulates linearly with increased cell.time. However, at the point of growth inhibition (red dashed line) the level is not inhibitory with reference to (C). Further, glucose and lactate specific rates do not show any notable change as growth becomes inhibited. (E) A screen of cytokines present in media after cell growth inhibition indicated TGF‐β1 as a primary candidate for feedback growth inhibition. (F) TGF‐β1 is shown to be slightly inhibitory to erythroblast cell growth with a maximum of 9% reduction in specific growth rate over 40 h of culture and 10 ng/ml TGF‐β1. (G) TGF‐β1 is produced at the same specific rate in static and bioreactor cultures.

Five hundred litres of media per unit of RBCs is still at least an order of magnitude below economic levels of intensification. Inhibition of cell growth by depletion of nutrients was tested by supplementation strategies of key media component groups including glucose, glutamine, serum, cytokines (EPO, SCF and IL‐3), amino acids, vitamins, and phosphate. However, this had no effect on the point at which growth inhibition occurred (Figure 6A, B). Further, only a low proportion of available glucose was depleted over the uninhibited growth period (Figure 6D); other key nutrients including iron, glutamine, and glutamate also showed negligible consumption rates over the period prior to growth inhibition (data not shown).

The alternative to medium depletion is production of an inhibitory factor such as lactate or ammonia (Hassell *et al*., 1991); addition of exogenous supplements of either significantly inhibited growth rate in a linear fashion (*p* ≤ 0.05; Figure 6C). The effect of each factor was dependent on the level of the other with high lactate levels reducing the inhibitory effect of ammonia. However, to cause the observed inhibition of growth ammonia/lactate combinations in excess of 4 mm/15 mm respectively would be necessary; accumulated concentrations of endogenously produced ammonia (ND i.e. <0.3 mm) and lactate (~6 mm) at the point of growth inhibition were much lower (Figure 6D). Additionally, after a brief initial higher period, the molar ratio of lactate produced to glucose used remained constant at 0.75 with no particular deviation associated with growth inhibition (Figure 6D). Finally, cultures were screened for the production of potentially inhibitory cytokines; TGF‐β, interferon‐γ and tumour necrosis factor‐α are prime candidates reported to inhibit erythroid growth; IL‐1‐β, IL‐2, IL‐4, IL‐6 and IL‐10 were also measured as potential feedback influences. TGF‐β1 was the only factor secreted at a relatively high (ng/ml) level (Figure 6E). Dosing of exogenous TGF‐β1 did decrease specific cell proliferative rate but only by 9% at 10 ng/ml, a higher dose level and lower inhibition than that observed in culture (Figure 6F). Although a substantive effect on proliferative rate was not observed, TGF‐β1 did accelerate erythroblast maturation: 1 ng/ml resulted in a faster increase in CD235a expression, earlier enucleation, and ~30% reduced total proliferative capacity of cells suggesting the cytokine may be responsible for the lower proliferation/higher enucleation in the bioreactor. However, the cell specific production rate of TGF‐β1 was equivalent in the static and the bioreactor system, showing a rapid decline in both systems over the first 5 h, after which it remained relatively stable (Figure 6G). Of further note, the TGF‐β1 was in inactive form (bound to latency associated peptide) in either static or bioreactor culture system (≤ limit of detection 1 pg/ml i.e. ≤0.1% of total).

## 4 Discussion

RBCs as a manufactured product will not become economically viable unless fundamental barriers to cell culture efficiency are identified and addressed. The work here has shown that the barriers conventionally associated with high intensity cell production are not the primary limitations for the field; on the contrary, erythroblast metabolic characteristics indicate that gas mass transfer requirements, nutrient use and metabolite resistance will allow high intensity production in current industry standard bioreactor systems. Further, certain system attributes, such as mechanical stress, can be advantageously controlled to increase product purity. This understanding is necessary to inform future research that will progress the manufactured RBC field. Any adoption of nonindustry standard bioreactors, or new bioreactor design, should be based upon specific requirements of the intensified process. Defining production limits in current commercial bioreactor systems is a key starting point; such systems lower the risks and barrier to entry for product developers due to regulatory and industrial experience.

Most cell cultures are limited in absolute density by O_2_ transfer into the system, and this will determine the minimum volumetric footprint for the manufacturing bioreactor. The low specific OUR of the erythroblasts is at least an order of magnitude beneath those reported for common cell lines (Ruffieux *et al*., 1998; Goudar *et al*., 2011). Even given the operational constraints on actively gassing and agitating the culture media this enables potentially very high intensity production. The frequency with which media needs to be exchanged to maintain uninhibited exponential growth is therefore the primary economic constraint. This does not necessarily force a large volume for the manufacturing bioreactor, but determines the total volume of medium used in a given production run. Allowing cells to drop significantly beneath uninhibited exponential growth is grossly time, and consequently cost, inefficient due to the compounding nature of cell doubling. The observed uninhibited growth rate potential is encouraging; a 13‐h erythroblast doubling time enables a 4‐order of magnitude increase in cell number in a week. However, the calculated rate of media exchange required to achieve this, with many minimally depleted factors wasted and common metabolites beneath toxic levels, is economically prohibitive.

A depleted medium factor or a secreted inhibitor could exhibit the same growth limiting behaviour observed. However, we have stronger evidence for the latter given the range of supplementary strategies that do not promote further cell growth. Further, the maintenance of a constant ratio of glucose consumption to lactate production suggests this is not a metabolic limitation; such limits would be likely to disrupt the ratio (Zagari *et al*., 2013). TGF‐β1 was present at high levels, and (as previously reported (Buscemi *et al*., 2011)), accelerated erythroblast maturation in a manner similar to that observed in the bioreactor when exogenously dosed in to static culture. The equivalent concentration and inactivity of the endogenous cytokine in both culture systems initially suggested it was an unlikely candidate for either growth rate inhibition or total reduced proliferation in the bioreactor. However, mechanical forces as low as 40 pN can transiently activate TGF‐β1 from its latent form; it is therefore reasonably probable that there is a bioreactor specific effect whilst stirring is applied causing accelerated maturation (Buscemi *et al*., 2011). Alternatively, or additionally, mechanical forces have been reported to have direct integrin mediated signalling effects that can influence cell maturation or inhibitory factor potency (Schwart, 2010). Although this could not explain the inhibition of proliferative rate (given the lack of substantive effect of TGF‐β1 dosing into the bioreactor on proliferation rate), other unidentified inhibitory mediators are likely to be secreted. Mechanical agitation has been reported to increase cytokine release and signalling in a number of other cell types so there is evidence that such factors could be present at higher levels, or more potent, in a stirred bioreactor (Kurazumi *et al*., 2011).

A further limit to RBC production *in vitro* is the red cell yield per starting progenitor cell; the nature of the limit is either availability or cost of the required starting cells. The contribution of the starting cells to the cost of a final RBC product depends on the proliferative capacity of the cells during differentiation – every order of magnitude in cell expansion (approximately 3.3 population doublings) achieved between starting cells and final product reduces the requirement for (and hence the impact of the cost of) the starting cells by an order of magnitude on a per product basis. Conversely, the impact on cost of the final product for production of a given cell phenotype becomes exponentially larger as the cells proliferate towards terminal differentiation i.e. 2 × 10^12^ terminally mature orthochromatic erythroblasts are required to make each unit of enucleated blood, but only ~2 × 10^8^ cells of the progenitor phenotype from ~14 PDs earlier in the process. This is important as differentiating cells have a changing profile of metabolism and other attributes that impact manufacturing productivity cost; in the case of red cells the potential to intensify would be anticipated to increase as the cells mature. The different approaches currently taken to overcome availability limitation of primary cells such as UCB – pluripotent, adult stem cell, engineered progenitor – will have different production costs that will be a function of cost of input cells and the subsequent proliferative capacity and intensification profile during differentiation; very recent progress to address both adult (vs. embryonic) maturation (Fujita *et al*., 2016) and yield (Giani *et al*., 2016) from renewable sources such as pluripotent cells has been promising. Our work has focused on erythroblast intensification because it will be a key determinant of process cost and practicality irrespective of the progenitor starting cell population due to both the exceptionally high number of these cells required in culture per unit of product and their proliferative capacity (Mercier Ythier, 2015). The data discussed here are therefore limiting and relevant for any candidate red cell manufacture process.

We conclude that there are no conventional barriers (shear stress sensitivity, O_2_ demand, or metabolic demand) that would prevent established bioreactor systems from producing blood at productivities under 100 l/unit, and possibly significantly higher. Further the effect of combined control of pH, oxygen, and mechanical agitation will greatly increase efficiency of final product harvest; in particular mechanical agitation, by rapidly increasing the proportion of enucleated cells, will enable peak enucleation to be engineered closer to peak culture system proliferation. This is absolutely key to reduce wastage of earlier enucleating cells, and to prevent challenging downstream processing of low purity enucleated product. However, the sensitivity of the cells to the bioprocess conditions adds risk and complexity as well as opportunity; mechanical stress may simultaneously increase enucleation whilst reducing total proliferative capacity, conventional biologics production strategies such as the addition of cell membrane protective agents appear to improve proliferation but reduce enucleation (presumably because membrane mechanics are critical for enucleation). To realize the potential efficiencies of production at suitably low risk, process scaling and intensification must be characterized for effects on all key elements of cell quality, and effort must be focused on identifying and mitigating the factor(s) that inhibit growth rate (and hence media efficiency).

## Key points

- Enucleated red cells can be produced to high purity in industry standard stirred tank bioreactors at 500 l per unit of cells
- Mass transfer and common metabolites are not primary limitations indicating potential for substantially higher efficiency

## Conflict of interest disclosures

No authors have any conflict of interest.
